# HIV-1 Antiretroviral Drug Resistance in Mozambique: A Systematic Review and Meta-Analysis

**DOI:** 10.3390/v16121808

**Published:** 2024-11-21

**Authors:** Paloma Gonçalves, Paulo Mascarenhas, Rute Marcelino, Nédio Mabunda, Arne Kroidl, W. Chris Buck, Ilesh Jani, Claudia Palladino, Nuno Taveira

**Affiliations:** 1 Research Institute for Medicines (iMed.ULisboa), Faculty of Pharmacy, Universidade de Lisboa, Avenida Professor Gama Pinto, 1649-003 Lisbon, Portugal; pcb.goncalves@campus.fct.unl.pt (P.G.); rute.marcelino@netcabo.pt (R.M.); cpalladino@edu.ulisboa.pt (C.P.); 2 Egas Moniz Center for Interdisciplinary Research (CiiEM), Egas Moniz School of Health & Science, Quinta da Granja, 2829-511 Caparica, Portugal; pmascarenhas@egasmoniz.edu.pt; 3 Instituto Nacional de Saúde de Mozambique (INS), Estrada Nacional N1, Maputo 1102, Mozambique; nedio.jonas@ins.gov.mz (N.M.); ilesh.jani@ins.gov.mz (I.J.); 4 Division of Infectious Disease and Tropical Medicine, University of Munich (LMU), Leopoldstraße 5, 80802 Munich, Germany; akroidl@lrz.uni-muenchen.de; 5 David Geffen School of Medicine, University of California Los Angeles, 10833 Le Conte Ave, Los Angeles, CA 90095, USA; wbuck@mednet.ucla.edu

**Keywords:** HIV drug resistance (HIVDR), antiretroviral therapy (ART), Mozambique, drug resistance mutations (DRMs), systematic review and meta-analysis

## Abstract

This systematic review assessed the prevalence of transmitted and acquired HIV drug resistance (HIVDR) and the associated risk factors in Mozambique. A search of the PubMed, Cochrane, B-On, and Scopus databases up to December 2023 was conducted and included 11 studies with 1118 HIV-1 pol sequences. Drug resistance mutations (DRMs) to NNRTIs were found in 13% of the drug-naive individuals and 31% of those on ART, while NRTI resistance occurred in 5% and 10%, respectively. Dual-class resistance (NNRTI + NRTI) was detected in 2% of the drug-naive and 8% of ART-experienced individuals. DRMs to protease inhibitors (PIs) were found in 2% of the drug-naive and 5% of ART-experienced individuals. The rate of DRMs was significantly higher in Beira than in Maputo, as well as in pediatric patients than in adults and pregnant women. Subtype C predominated (94%) and was associated with lower viral loads and DRM rates as compared to the other subtypes. The high prevalence of DRMs, particularly to NNRTIs and NRTIs, highlights the need for ongoing surveillance and targeted interventions. These findings are critical for optimizing ART regimens and informing public health strategies in Mozambique, with particular attention to regions such as Beira and vulnerable populations such as pediatric patients.

## 1. Introduction

In 2022, 2.4 million adults and children were living with HIV in Mozambique (12.4%) [1,2,3]. HIV prevalence in Mozambique is higher in women (15.2%) than in men (9.5%) [3,4]. Since the first AIDS case report in 1986, the country has faced three major phases of the HIV incidence trend. A steady increase until 2001, with up to 150,000 new infections per year, a plateau trend over the following decade, and a decreasing trend thereafter, reaching 94,000 new cases in 2021 [4]. AIDS-related deaths peaked in 2006 (73,000) and decreased to about 35,463 deaths in 2021 [2]. The epidemic is mainly driven by HIV-1 subtype C [5,6,7,8,9,10,11,12,13,14,15,16,17].

Since 2004, free antiretroviral therapy (ART) has been available in Mozambique, based on the World Health Organization (WHO) public health approach to ART delivery [18,19]. In the early stages of ART roll-out, adults were treated with the first-line fixed dose combination of a non-nucleoside reverse transcriptase inhibitor (NNRTI), nevirapine, and two NRTI, stavudine/lamivudine (d4T/3TC/NVP). Later, first-line therapy consisted mainly of the NNRTI efavirenz (EFV)-based therapy, mostly with tenofovir (TDF) and 3TC [18,19,20]. However, in 2020, the high rate of resistance (50% to 97%) [5,11,14,16,21,22,23,24] led to a shift to a dolutegravir (DTG)-based first-line regimen with two NRTIs as an optimized backbone [25,26]. Second-line ART regimens consist of a protease inhibitor (PI) and two NRTIs [20]. For prevention of mother-to-child transmission (PMTCT), recent guidelines recommend DTG+3TC+TDF for mothers and AZT+NVP dual syrup prophylaxis for all exposed infants [27]. In the early stages of ART roll-out, pediatric patients were treated with d4T/3TC/NVP. Later, most children were on AZT/3TC/NVP until a PI-based first-line regimen for younger children with boosted lopinavir (LPV/r) was introduced in 2018. The massive switch to DTG in children occurred in 2022 with the introduction of the pediatric formulation pDTG (10 mg) plus abacavir/lamivudine (ABC/3TC).

Generalized antiretroviral therapy has reduced HIV transmission and mortality in Mozambique but, as in the rest of the world, it has increased the prevalence of drug resistance [2,3,24]. A recent survey of drug-naive subjects showed that the northern region of Mozambique had the highest prevalence of transmitted drug resistance (TDR) (8.5%, 95% CI: 4.9; 14.3) compared with the southern (6.4%, 95% CI: 3.7; 10.2) and central (3.9%, 95% CI: 3.9; 9.7) regions [16]. The most common NNRTI TDR mutations are K103N, E138A, V179D, and G190A, and M184V and thymidine analogue mutations (TAMs) (M41L, D67N and T215A) are the most common mutations associated with resistance to NRTIs.

The risk of developing drug resistance in subjects on ART in Mozambique is high due to low uptake of VL monitoring (64% in 2022), drug resistance testing [27], and low virologic suppression rates [28]. The country has one of the lowest levels of trained nurses and doctors in the world, with only 0.04 doctors and 0.41 nurses/midwives per 1000 people [29]. Between 2005 and 2021, the coverage of health facilities with ART services in the country increased from 1% to 96% [27]. However, retention in HIV care and treatment remains a major challenge, with studies have reported a 66% retention at 12 months, dropping to 52% at 24 months, and then to 44% at 36 months [19,27,30]. Poor retention in HIV care has been linked to several factors, including stigma and discrimination, poverty and unemployment, work/childcare responsibilities, distance and transport to the health facilities, side effects of ART medication, the complexity of dosing schedules, and the reliance on traditional medicines [30,31,32,33].

This systematic review and meta-analysis aims to provide a consolidated understanding of the prevalence, trends, and patterns of HIV drug resistance (HIVDR) in Mozambique and to assess the associated risk factors. Close monitoring of drug resistance aids in informing healthcare policies, guiding treatment strategies, and identifying areas for further research or intervention to effectively combat HIV/AIDS in Mozambique.

## 2. Materials and Methods

This review was conducted in accordance with the Preferred Reporting Items for Systematic Reviews and Meta-Analyses (PRISMA) guidelines [34], and its protocol was registered on the PROSPERO international prospective register of systematic reviews of the National Institute for Health Research (Ref. ID: CRD42022327228).

### 2.1. Electronic Databases

The following four databases were searched for relevant studies: PubMed, B-on, Scopus, and the Cochrane Library. We supplemented the database searches by manually screening the bibliographies of systematic reviews in the Cochrane database.

### 2.2. Search Strategy for Study Identification

A broad search strategy was used, combining ART, HIVDR, and HIV infection terms and text words with Medical Subject Heading (MeSH) terms. The literature search algorithm was designed to identify studies that reported HIVDR data among PLWH in Mozambique since the first AIDS case was reported in 1986. The search was conducted between May 2022 and 31 December 2023. The bibliographies of retrieved studies were reviewed to identify additional papers. The search terms used are listed in Table S1.

### 2.3. Study Selection and Inclusion Criteria

Three authors (RM, PG, and CP) reviewed the studies. If different reports were based on the same original trials or observational studies and there was data redundancy, only one report was included in the review. However, the excluded reports were still screened for additional information on study population characteristics that may not have been included in the selected study report. We included any original study on PLWH in Mozambique reporting HIVDR data. We did not have any language or publication status restrictions. For reviews, we checked their bibliographies for original studies. We excluded studies that did not include original data; studies with less than ten participants; studies conducted in a migrant population; and studies that did not report GenBank accession numbers for pol gene sequences.

### 2.4. Data Extraction

To ensure data quality (consistency and accuracy), study level and patient data were systematically extracted. The following study-level data were extracted as they may influence HIVDR: study location, year of sample collection, age, sex, pregnancy status, CD4+ T cell count (cells/mm^3^), and HIV viral load. Additionally, exposure to antiretroviral (ARV) drugs before treatment initiation was considered (yes, no, or unknown).

### 2.5. Sources of HIV Sequences and Identification of DRMs

HIV-1 nucleotide sequences were retrieved from GenBank (NCBI) using the accession numbers provided in the selected articles. A complete list of these accession numbers is provided in Table S2. HIV-1 subtypes were confirmed by phylogenetic analysis using the maximum likelihood (ML) method with 1000 bootstrap replicates in the IQ-tree 1.6.8 software [35] and visualized using FigTree V1.4.3 [36]. The best-fitting partitioning scheme and nucleotide substitution model were selected based on the lowest Bayesian Information Criterion (BIC) values as implemented in IQ-tree. Bootstrap values >70% were considered definitive for significant clustering.

Resistance-associated mutations in PR, RT, and IN coding regions and resistance profiles were identified using the Stanford HIVdb algorithm version 9.3 [7,37,38].

### 2.6. Statistical Analyses

Categorical variables were reported as percentages, while continuous variables were presented as medians with interquartile range (IQR) or means with standard deviations (SDs). The prevalence of mutations was compared between the study groups (drug-naïve and those receiving antiretroviral therapy) using Fisher’s exact test. All meta-analysis (MA) statistics and associated plots were performed using the Open Meta Analyst software, version 3.9 [39]. Four distinct outcomes were calculated as pooled effect sizes from the data extracted from the selected articles. The primary outcome was the drug resistance mutation rate per sequence (DRMR), defined as the ratio of the total number of resistance mutations to the total number of sequences. The secondary effect sizes were the relative mutation risk ratio (MRR), derived from the DRMR, and, when available in the selected studies, the mean plasma viral load (log10 HIV RNA copies/mL) and the CD4+ T-cell count (cells/mm^3^). The MRR was calculated as the ratio between the ART and drug-naive subjects’ DRMR meta-regression exponentiated coefficients. All MA overall results were estimated using restricted maximum likelihood random effects models and reported with 95% confidence intervals (95% CI). The overall results from all meta-analyses were evaluated for homogeneity using the I^2^ heterogeneity index. The chi-squared (χ^2^) test for heterogeneity was employed to ascertain the degree of homogeneity, classified as high or low [40]. Z-tests for proportions or means were employed, where appropriate, to ascertain whether the MA outcomes were significant and associated with some effect. The same Z-tests were employed to compare the MA outcomes between the study groups. Furthermore, meta-regressions were conducted to ascertain the potential sources of heterogeneity in the meta-analysis. The study population meta-regression covariates evaluated were, where available, antiretroviral therapy exposure, mean age, male-to-female ratio, sample type from which HIV-1 sequences were obtained, participant group (adults, neonates/children, or pregnant women), study region, and HIV subtype C ratio. The fitted parameters of the meta-regressions included coefficients for each covariate that quantified the correlational effect of the variable(s) of interest on the meta-analytic outcome. The combined impact of the study population covariates was assessed whenever the meta-regression model coefficients yielded significant Z-test results. The proportion of the overall outcome variance explained by the variables in the meta-regression was defined by R^2^. For all statistics, the significance was defined as a two-tailed *p*-value of less than 0.05.

### 2.7. Risk of Bias Assessment

The internal validity (risk of bias) of the included studies was evaluated using the Joanna Briggs Institute (JBI) critical appraisal checklist for studies reporting prevalence or incidence data [41]. The tool comprises nine questions with four possible answers, each assigned a score of 1 (yes), 0 (no), or 0 (unclear or not applicable). The obtained score is presented in percentages that categorize each study according to the different levels of risk of bias, as follows: high (20–50%), moderate (50–80%), or low (80–100%). The Risk-Of-Bias VISualization (ROBVIS) tool was employed to generate risk-of-bias plots [42]. Two authors (RM and PG) evaluated the quality of the studies, and any discrepancies were resolved through discussion with a third author (PM).

## 3. Results

### 3.1. Study Selection

**Figure 1:**
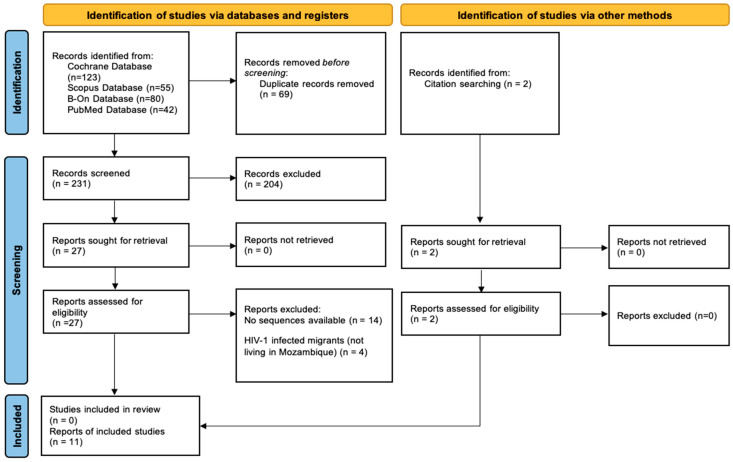
The PRISMA flow chart summarizing the process of identifying, screening, and including relevant studies (n = 11) based on the specified inclusion and exclusion criteria.

A total of 302 studies were identified through the search process, of which 69 were duplicates. After removing the duplicates (n = 69), 204 studies were excluded after reviewing the title and/or abstract. From the overall 29 entries included for full article review eligibility, 14 did not report GenBank accession numbers for pol gene sequences, and 4 were conducted in a migrant population. This process led to the inclusion of 11 studies in the final review [5,6,8,9,10,11,13,14,15,23,43]. The PRISMA flow diagram, which provides a summary of the process of identification, screening, and inclusion of the relevant studies based on the inclusion and exclusion criteria, is presented in Figure 1.

### 3.2. Studies Characteristics

The main characteristics of the studies included in this review are presented in Table 1. All studies were observational and conducted in the cities of Maputo, Beira, Nampula, and Pemba. A total of 152 pediatric subjects, comprising neonates, infants, and children, were included in two studies. Pregnant women (363) were included in three studies, while adults (591) were included in the remaining six studies. The age of the subjects ranged from 0 to 63 years old. The adult cohort comprised 278 women and 254 men. The sex of the remaining 59 adult participants was not reported in the studies. A total of 837 subjects (75%) were drug naive. The remaining 281 subjects (25%) were on NNRTI-based ART with a 2-NRTI backbone, specifically AZT (or d4T) + 3TC + NVP (or EFV). One study employed NVP (postnatal) + AZT (first week of life).

The studies encompassed a total of 1118 HIV-1 polymerase (pol) gene sequences derived from viruses present in plasma, breast milk, and cells. A total of nine studies analyzed reverse transcriptase and protease sequences, while one study focused on integrase sequences. All sequences were subjected to phylogenetic and drug resistance genotyping analysis. Phylogenetic analysis revealed that 94% (1055/1118) of the sequences were classified as subtype C, 2% (25/1118) as subtype A, 1% (16/1118) as subtype G, 1% (10/1118) as subtype D, and 0.2% (2/1118) as subtype B. Circulating recombinant forms (CRF37_cpx and CRF41_CD) were observed in 1% (9/1118) of the sequences, while unique recombinant forms were identified in 0.1% (1/1118). The nine studies that analyzed amino acid sequences of the reverse transcriptase enzyme identified mutations that confer resistance to NRTIs and NNRTIs.

### 3.3. Risk of Bias Within Studies

**Figure 2:**
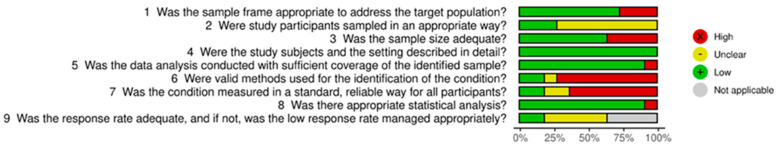
Summary of the evaluated risk of bias domains for the included studies, according to the percentage of the scores provided by the Joanna Briggs Institute tool.

All the studies provided a clear description of their subjects and settings (question 4 in Figure 2). A majority of the studies had an appropriate sample frame to address the target population (n = 8, 73%) (question 1), justified the sample size (n = 7, 63%) (question 3), conducted a data analysis with sufficient coverage of the identified sample (n = 10, 91%) (question 5), and provided an adequate statistical analysis (n = 10, 91%) (question 8 in Figure 2). However, several studies significantly failed to provide information on the sample selection criteria (n = 8, 73%) (question 2), a clear description of the study protocol (n = 9, 82%) (question 6), a clear report of the experimental protocol (n = 9, 82%) (question 7), and a complete response rate analysis (n = 9, 82%) (question 9).

### 3.4. Results of the Meta-Analysis

In drug-naive subjects, the DRM rate (DRMR) was lower in Maputo (0.095; 95% CI: 0.024; 0.207; I^2^ = 91.4%) than in other cities, especially when compared to Beira, which had the highest DRMR (0.535; 95% CI: 0.386; 0.680) (Table 2 and Figure S1). The DRMR was similar in the pregnant women (0.166; 95% CI: 0.077; 0.281; I^2^ = 85.4%) and adult subjects (0.169; 95% CI: 0.048; 0.344; I^2^ = 94.6%). Finally, the DRMR determined in plasma viruses of these subjects was found to be lower (0.126; 95% CI: 0.056; 0.218; I^2^ = 90.0%) than the DRMR determined in integrated proviral DNA (cell viruses) (0.367; 95% CI: 0.105; 0.683; I^2^ = 92.5%).

Among those receiving antiretroviral therapy (ART), the DRMR values were also lower in Maputo (having plasma viruses as source: 0.318; 95% CI: 0.059; 0.664; I^2^ was 96.3) and higher in Beira (having cells as source: 0.848; 95% CI: 0.708; 0.948). It is noteworthy that neonates, infants, and children on ART exhibited a higher DRMR (0.747; 95% CI: 0.533; 0.912; I^2^ = 81.7%) compared to adults and pregnant women (Table 3 and Figure S2).

No treatment group (drug-naive individuals) was associated with a lower DRMR (0.169; 95% CI: 0.082; 0.280; I^2^ = 93.3%) (Table 4 and Figure S3). Infants treated with NVP (after birth) + AZT (first week of life) had the highest DRMR compared to the other treatments (0.848; 95% CI: 0.708; 0.948), followed by individuals treated with AZT (or d4T) + 3TC + NVP (or EFV) (0.652; 95% CI: 0.561; 0.737). Individuals treated with AZT (or d4T) + 3TC + NVP (or EFV) and drug-naive individuals had similar levels of DRMR (Table 4).

The combination of AZT (or d4T) with 3TC and NVP (or EFV) in adults and NVP (after birth) with AZT (in the first week of life) was associated with a 2.9-fold and 3.8-fold relative increase in DRMR risk, respectively, compared to no ART (drug-naive individuals) (Table 5).

Potential confounders of DRMR, including characteristics of the study population and some individual-level predictors, were examined by meta-regression. Subtype C (the predominant type) was associated with a lower DRMR than the average DRMR for all the other subtypes combined (*p* < 0.0012) (Table 6). The AZT (or d4T) + 3TC + NPV (or EFV) regimen was associated with significantly elevated DRMR compared to the AZT/d4T + 3TC + NVP regimen (*p* = 0.0011). Regarding the origin of the sequenced viruses, the results show a significant increase in DRMR for the viruses from cells compared to the plasma viruses (*p* < 0.0001). The pooled results show a significantly lower DRMR associated with Maputo as compared to the other regions (Figure S1 and Table 2); however, when this rate is adjusted for other covariates (male %, subtype C, treatment, HIV viral population and patient), Nampula/Pemba takes the lead (Table 6).

We also performed a meta-analysis to analyze the influence of different predictors on viral load (VL). Drug-naive individuals were associated with a 1.4-fold increase in VL as compared to treated individuals (Table 7 and Figure S4).

HIV-1 subtype C infection was associated with a lower VL as compared to the other subtypes combined (*p* < 0.001) (Table 8, model 1). In the same model, ART consisting of AZT (or d4T) + 3TC + NVP was associated with a decrease in VL as compared to that of the drug-naive subjects (*p* < 0.001). In model 2, the meta-regression analysis showed no confounding variables related to the participant group (Table 8). Finally, ART consisting of AZT (or d4T) + 3TC + NVP was associated with an increase in mean CD4+ T-cell count (*p* < 0.001) compared to the drug-naive subjects (Table 9).

### 3.5. Genotypic Drug Resistance Analysis

**Figure 3:**
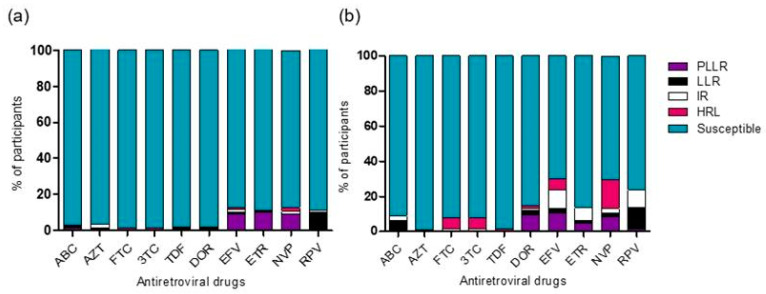
Susceptibility and resistance to NNRTIs and NRTIs in drug-naive subjects (**a**) and subjects on ART (**b**). HIVDR was predicted using the Stanford HIVdb algorithm score: Susceptible (0–9); Potential Low-Level Resistance (PLLR) (10–14), Low-Level Resistance (LLR) (15–29), Intermediate Resistance (IR) (30–59), and High-Level Resistance (HLR) (>60). NRTIs: ABC, Abacavir; AZT, Zidovudine; FTC, Emtricitabine; 3TC, Lamivudine; TDF, Tenofovir. NNRTIs: DOR, Doravirine; EFV, Efavirenz; ETR, Etravirine; NVP, Nevirapine; RPV, Rilpivirine.

Genotypic analysis of the 1118 sequences included in the study showed that 2.0% (17/837) of drug-naive subjects and 7.8% (22/281) of those receiving ART had at least one mutation conferring resistance to RT inhibitors. Approximately 12.7% (106/837) of drug-naive participants and 31.0% (87/281) of those receiving ART had DRMs to NNRTIs. Between 11% and 13% of drug-naive subjects had reduced susceptibility to EFV, ETR, NVP, and RPV (Figure 3a). DRMs to NVP and EFV were identified in 11.9% (100/837) of the drug-naive subjects and 29.9% (84/281) of those receiving ART. Specifically, 16.7% (47/281) of participants receiving ART had a high-level resistance to NVP and 6% to EFV (Figure 3b). Up to 23.5% (66/281) of participants on ART had low-level resistance (LLR) and intermediate-level resistance (IR) to RPV, and approximately 14.9% (42/281) had reduced susceptibility to DOR and ETR. The most common NNRTI resistance mutations were K103N (naive, n = 8/837, 1.0%; treated, n = 11/281, 3.9%), E138A/G (naive, n = 67/837, 8.0%; treated, n = 20/281, 7.1%), Y181C/H/L (naive, n = 4/837, 0.5%; treated, n = 21/281, 7.5%), and G190A (naive, n = 5/837, 0.6%; treated, n = 8/281, 2.8%) (Table S3).

Approximately 4.5% (38/837) of drug-naive subjects and 9.6% (27/281) of those receiving ART had mutations conferring resistance to NRTIs. The most common mutation was M184I/T/V (naive, n = 9/837, 1.1%; treated, n = 17/281, 6.0%) (Table S3). Approximately 7.8% (22/281) of those receiving ART had resistance mutations to FTC and 3TC, of which 6.0% (17/281) conferred a high-level resistance (Figure 3b). Drug-naive participants had high levels of susceptibility to all NRTIs (Figure 3a). Approximately 8.9% (25/281) of participants on ART had low or intermediate resistance to ABC (Figure 3b).

Although none of the 281 participants on ART had been treated with PIs, 4.6% (13/281) had at least one mutation conferring PI resistance. Approximately 2.4% (20/837) of the sequences belonging to drug-naive individuals also had PI resistance mutations. Only 0.2% (2/837) of drug-naive individuals and 0.7% (2/281) of individuals receiving ART had mutations associated with resistance to INSTI. All mutations are listed in Table S3. Some mutations conferring resistance to NNRTIs, NRTIs, and PIs were significantly more common in the subjects on ART as compared to the untreated subjects (Table 10).

## 4. Discussion

We conducted the first systematic review and meta-analysis of the prevalence of transmitted drug resistance and acquired HIVDR in Mozambique and assessed the risk factors associated with the emergence of HIVDR. Overall, our analysis showed that 2% of the drug-naive individuals and 8% of those on ART had at least one mutation conferring resistance to reverse transcriptase inhibitors (NRTI or NNRTI). The prevalence of NNRTI resistance was 13% in the drug-naive subjects and 31% in the subjects on ART. The most recent WHO survey of drug resistance in Africa reported an average prevalence of transmitted NNRTI resistance of 11%, which is similar to our findings [24]. However, the prevalence of acquired NNRTI resistance (i.e., in treated individuals) is higher across Africa than in Mozambique, ranging from 50% to 97% [22,24,44,45,46,47]. The prevalence of NNRTI resistance among people on ART in Mozambique is likely to be underestimated, as the most recent survey on this issue in Mozambique dates back to 2014.

The most prevalent NNRTI resistance mutations found in our study were K103N, Y181C, G190A (major mutations), and E138A (accessory mutation), which confer high- or intermediate-level resistance to NVP and EFV, the most commonly used NNRTIs in first-line ART regimens in Africa [22,24]. Y181C is of concern because it also confers resistance to the newer generation of NNRTIs, such as etravirine (ETR) and rilpivirine (RPV) [37].

The prevalence of resistance to NRTIs was estimated to be 5% among the drug-naive subjects, which is close to the mean prevalence reported in the WHO survey (3%) [24] and in Mozambique’s neighboring countries (ranging from 1% in Zambia, Zimbabwe, and Eswatini to 8% in Tanzania) [24,44,47,48,49,50,51]. However, the mean prevalence of an acquired resistance to NRTIs was much lower than that reported in the WHO survey (10% vs. 48%) [24] and in Mozambique’s neighboring countries (ranging from 53% in Eswatini to 68% in Zambia) [24,44,45,46,47]. As with NNRTI resistance, the NRTI results in Mozambique are also likely to be underestimated, as the most recent survey of NRTI drug resistance in treated individuals also dates back to 2014. As in the previous studies, the most common NRTI resistance mutation in our dataset was M184V (n = 26, 7%) [24,44,46,47,48,49,50,51,52,53,54]. The low prevalence of TAMs and K65R and the absence of multidrug resistance mutations (T69ins, Q151M) are in contrast to the previous studies and probably do not reflect the current situation.

In our study, less than 1% of subjects had resistance mutations for INSTIs, which, when combined with the high prevalence of NNRTI resistance, highlights the appropriateness of moving to DTG-based regimens as the preferred first-line regimen for PLWH initiating ART, in line with WHO recommendations [26]. DTG is a second-generation INSTI with significant public health benefits due to its availability as a low-cost fixed-dose combination of TDF, 3TC, and DTG and its high genetic barrier to resistance [55,56,57]. Few major PI resistance mutations were detected in this study, supporting the continued use of PI-based regimens as an alternative therapeutic regimen in cases of virologic failure.

Among subjects on ART, the DRMR was higher in pediatric participants compared to pregnant women and adults (3.4-fold and 6.7-fold, respectively), and infants treated with NVP (after birth) + AZT (first week of life) had the highest risk ratio for a higher DRMR. These findings are consistent with the low effectiveness of the current NVP-based PMTCT regimens used in sub-Saharan Africa and provide further support for a rapid transition to DTG-based regimens [25,55,56,57,58,59,60,61,62].

Living in Beira was associated with the highest DRMR among both people on ART and drug-naive subjects, which is consistent with the data reporting that Sofala, of which Beira is the capital, is the province with the lowest uptake of viral load monitoring and drug-resistance testing in Mozambique [27].

A higher DRM rate was found in the proviral DNA than in the plasma viral RNA of both the drug-naive and treated individuals. Cell-associated proviral DNA archives wild-type and drug-resistant viruses for months to years, even in the absence of therapy [63,64,65]. In contrast, the selection and maintenance of plasma viruses with DRM requires an exposure to antiretroviral drugs because, in the absence of therapy, the drug-resistant viruses are easily outcompeted by wild-type viruses or mutate back to their wild-type form [63,64,66,67,68,69,70]. Therefore, the lower DRM rate found in viral RNA in this study may be a consequence of low adherence to ART and poor retention in HIV care in this population [63,64].

HIV-1 subtype C accounted for 94% of the HIV-1 infections in our dataset, but other subtypes (A, B, D, G) and recombinant forms were also found, suggesting a dynamic epidemic with multiple sources. Natural polymorphisms that occur in different HIV-1 subtypes can influence antiretroviral drug susceptibility, the extent of resistance, and the propensity to acquire some DRMs [71,72,73,74,75,76]. In a recent global clinical trial, a subtype C infection was associated with a shorter time to virological failure on antiretroviral treatment as compared to subtype B, and an infection with non-B-non-C subtypes was associated with a longer time to failure [77]. In our dataset, subtype C infection was associated with a lower viral load and a lower DRMR compared to the other subtypes, suggesting better treatment management of subtype C-infected subjects compared to non-C-infected subjects.

This review has several limitations. First, despite using a broad and highly sensitive search strategy and searching several academic research databases, only a limited number of studies conducted in Mozambique were available for inclusion. The studies in question provided rather limited and simplified information and failed to include some crucial details such as the ethnicity of the participants, the mode of transmission, the duration of HIV-1 infection, the duration of treatment and the level of adherence. Second, all the studies included in the meta-analysis were of low or moderate quality. Most studies lacked inclusion criteria for participants, a clear description of the selection and experimental protocol, and an analysis of the response rate. These issues are important to minimize selection bias and strengthen the generalizability of the results. Third, our meta-analysis showed high heterogeneity among the included studies. The variation between studies may be due to the lack of methodological homogeneity and information (mentioned above), which could have influenced the overall results of each study.

## 5. Conclusions

This systematic review and meta-analysis highlight significant concerns regarding HIV drug resistance (HIVDR) in Mozambique, particularly among patients receiving antiretroviral therapy (ART). The findings indicate a considerable prevalence of drug resistance mutations (DRMs), especially to NNRTIs and NRTIs, which underscores the critical need for continuous surveillance and tailored interventions. The notably higher DRM rates in Beira as compared to Maputo, as well as among the pediatric groups as compared to the adults and pregnant women, point to geographical and demographic disparities that warrant targeted strategies. The predominance of subtype C, associated with lower viral loads and DRMRs, suggests the potential subtype-specific differences in drug resistance that should be considered in treatment protocols. These insights are crucial for optimizing ART regimens, improving patient outcomes, and informing public health policies in Mozambique to effectively manage and mitigate HIVDR.

## Figures and Tables

**Table 1:** Study level data from included studies (n = 11).

Authors [Year of Publication]	Information								Main Findings	Funding
	No. Sequences	Sample Collection Date	HIV Viral Population from	Participants	Age in Years [Mean (SD); Median (IQR) or Range)]	Male/ Female	Region	Antiretroviral Drugs		
Andreotti et al. (2009) [10]	101	2009	Plasma Breast milk PBMC	Pregnant	[26.2 (+/−4.5); 26 (17–39)]	0/53	Maputo	AZT (or d4T) + 3TC + NVP	Almost all strains were subtype C (exceptions: 1 subtype A and 1 subtype G). DRMs: ■ NRTI: M184I/V ■ NNRTI: K103N, V106A and V108I	Drug Resource Enhancement against AIDS in Mozambique (DREAM Program)
Oliveira et al. (2012) [13]	57	December 2009 to August 2010	Plasma	Adult	>18 (NA)	18/39	Maputo	AZT (or d4T) + 3TC + NVP	92.9% of sequences were subtype C (exceptions: 1.7% subtype A and 5.4% URF_CG). DRMs: ■ INSTI accessory: T97A (1.8%) and E157Q (1.8%)	Brazilian National Research Council, (471299/2009-0) and National AIDS Program/Ministery of Health, Brazil, and Universidade Federal do Rio de Janeiro (UFRJ), Brazil
Lahuerta et al. (2008) [9]	51	1999 and 2004	Plasma	Adult	Means: 33 (IQR 25–48) and 23 (IQR 20–30)	0/81	Maputo	Naive	All sequences were subtype C. No DRMs.	Spanish Ministry of Education and Science (SAF-05845) (BFU2006-01066/BMC) (FIPSE project 36549/06); Vizcaya Argentaria foundation (BBVA 02-0); Spanish Fondo de Investigación Sanitaria (FIS01/1236).
Micek et al. (2014) [43]	33	Between June 2005 and May 2008	PBMC	Neonates/Infants	0–2 (NA)	NA	Beira	NVP (after birth) and AZT (first week of life)	All sequences were subtype C. NVP resistance was detected in 37.5% of infants infected via breast milk. DRMs: ■ NRTI: M41L and M184V ■ NNRTI: K103N, Y181C and G190A	National Institutes of Health (R01 AI058723 and STD/AIDS T32 AI07140) and the University of Washington/Fred Hutch Center for AIDS Research (CFAR) (P30 AI027757).
Bila et al. (2013) [14]	112	March to June in 2007 and in 2009	PBMC	Pregnant	15–25 (NA)	0:234	Maputo and Beira	Naive	All sequences were subtype C. TDR was classified as <5% in Maputo and 5–15% in Beira. DRMs: ■ NRTI: M41L and M184V ■ NNRTI: K103N, E138A/G, V179E and Y181H. ■ PI accessory: Q58E	NA
Vubil et al. (2016) [15]	95	November 2009 and June 2010	Plasma	Adult	29 (NA)	120:0	Nampula and Pemba	Naive	80% of strains were subtype C (exceptions: 10.5% subtype A, 3.2% subtype D and 2.1% subtype G). DRMs: ■ NRTI: K219E ■ NNRTI: K101E, K103N and G190A ■ PI accessory: L10I/F	Instituto Nacional de Saúde, Mozambique; Fundo Nacional de Investigação, Mozambique (IOC-FIOCRUZ)
Bellocchi et al. (2005) [5]	58	First months of 2003	Plasma	Adult	NA	-	Maputo	Naive	All virus strains were subtype C. DRMs: ■ NNRTI: E138A and V170D/E ■ PI major: I50L ■ PI accessory: V32G	Drug Resource Enhancement against AIDS in Mozambique (DREAM Program)
Parreira et al. (2006) [6]	43	August and October 2003	PBMC	Adult	Mean: 28.8 (1–63)	34:96	Beira	Naive	All virus strains were subtype C. DRMs: ■ NRTI: K70Q/R, V75A, F116Y and M184I/T ■ NNRTI: A98G, K103N, E138A, V179D, Y181C and P236L ■ PI major: I84V ■ PI accessory: M46V, N83D and L89V	Fundacão GlaxoSmithKline das Ciências de Saúde, Portugal
Bártolo et al. (2009) [11]	186	Between 2002–2004	Plasma	Adult	Mean: 41 (+/−12)	82:62	Maputo	Naive	Most strains were to subtype C (exceptions: 3.8% subtype G, 6.7% CRF37_cpx and 7.7% of other CRFs). DRMs: ■ NRTI: M41L, D67N, K70R, M184V, L210W, T215F/Y, K219Q ■ NNRTI mutations: A98G, K1001E, K103N, E138A, V179T, Y181C, G190A, P225H ■ PI major: M46I and L90M ■ PI accessory: K20T, Q58E and T74P	NA
Vaz et al. (2012) [23]	112	October 2007 and June 2008	Plasma	Infants/Child	Median: 2 (1.3–5.9)	43:76	Maputo	ZDV or d4T + 3TC + NVP (or EFV)	All virus strains were subtype C. DRMs: ■ NRTI: A62V, K65R, K70R and L74I/V ■ NNRTI: A98G, M184I/V, K219E, K101E, K103N, V106A/M, V108I, E138A/G, V179D, Y181C, Y188F, G190A and H221Y ■ PI accessory: K43T and Q58.	The Bill & Melinda Gates Foundation (K23 AI074423-05) European Community’s Seventh Framework Programme (FP7/2007–2013)
Abreu et al. (2008) [8]	76	2002	Plasma	Pregnant	NA	-	Multicenter	Naive	Northern: 64.3% of isolates were subtype C (exceptions: 18% subtype A, 11% subtype D and 7.1% others CRFs). Southern: 95% isolates were subtype C (exceptions: 5% subtype D). Central: 100% of isolates were subtype C. DRMs: ■ NNRTI: V108I, E138A/G, V179D and Y181C. ■ PI accessory: L10F, I50F and I84M.	Programa de Cooperação em Ciência, Tecnologia e Inovação com Países da África-PROAFRICA (Proc# 491367/2005-8) with Federal University of Rio de Janeiro, Rio de Janeiro, Brazil

NA—Not available; AZT—Zidovudine; d4T—Stavudine; 3TC—Lamivudine; NVP—Nevirapine; EFV—Efavirenz; SD—Standard deviation; IQR—Interquartile range; DRM—Drug resistance mutations; TDR—Transmitted drug resistance; PBMC—Peripheral blood mononuclear cells; PI—Protease inhibitor; NRTI—Nucleoside/nucleotide reverse transcriptase inhibitors; NNRTI—Non-nucleoside reverse transcriptase inhibitors.

**Table 2:** Meta-analysis of drug resistance mutation rate (DRM Rate) from all studies in drug-naive subjects.

Variable	No. Studies	Total No. of DRMs/Total No. of Sequences	DRM Rate (95% CI) ^a^	Heterogeneity Index I^2^ (%)
**Localization:**				
Maputo	5	78/511	0.095 (0.024; 0.207)	91.4 *
Maputo/Beira	1	25/112	0.233 (0.151; 0.305)	NA
Nampula/Pemba	1	19/95	0.200 (0.126; 0.286)	NA
Beira	1	23/43	0.535 (0.386; 0.680)	NA
Multicentric	1	17/76	0.224 (0.138; 0.324)	NA
**Sequence origin:**				
Plasma	7	114/682	0.126 (0.056; 0.218)	90.0 *
Cells	2	48/155	0.367 (0.105; 0.683)	92.5 *
**Participant’s group:**				
Pregnant women	3	56/364	0.166 (0.077; 0.281)	85.4 *
Adults	6	106/473	0.169 (0.048; 0.344)	93.2 *

^a^ Drug resistance mutation (DRM) rate—ratio of the total number of DRMs to the total number of sequences; * Chi-squared *p*-value < 0.01 for heterogeneity (H0: I^2^ > 50%); CI—Confidence Interval; NA—Not applicable.

**Table 3:** Meta-analysis of drug resistance mutation rate (DRM Rate) from all studies in subjects on ART.

Variable	No. Studies	Total No. of DRMs/Total No. of Sequences	DRM Rate (95% CI) ^a^	Heterogeneity Index I^2^ (%)
**Localization (sequence origin):**				
Maputo (Plasma)	3	101/248	0.318 (0.059; 0.664)	96.3 *
Beira (Cells)	1	28/33	0.848 (0.708; 0.948)	NA
**Participant’s group:**				
Pregnant women	1	26/118	0.220 (0.151; 0.299)	NA
Adults	1	2/18	0.111 (0.012; 0.292)	NA
Neonates/Infants/Children	2	101/145	0.747 (0.533; 0.912)	81.7 *

^a^ Drug resistance mutation (DRM) rate—Ratio of the total number of DRMs to the total number of sequences; * Chi-squared *p*-value < 0.01 for heterogeneity (H0: I^2^ > 50%); CI—Confidence Interval; NA—Not applicable.

**Table 4:** Meta-analysis of the drug resistance mutation rate (DRM Rate) according to treatment regimen.

Treatment Regimen	No. Studies	Total No. of DRMs/Total No. of Sequences	DRM Rate (95% CI) ^a^	Heterogeneity Index I^2^ (%)
Drug-naive	9	162/837	0.169 (0.082; 0.280)	93.3 *
AZT (or d4T) + 3TC + NVP	2	28/136	0.192 (0.105; 0.298)	27.7
NVP (after birth) + AZT (first week of life)	1	28/33	0.848 (0.708; 0.948)	NA
AZT (or d4T) + 3TC + NVP (or EFV)	1	73/112	0.652 (0.561; 0.737)	NA

^a^ Drug resistance mutation (DRM) rate—ratio of the total number of DRMs to the total number of sequences; * Chi-squared *p*-value < 0.01 for heterogeneity (H0: I^2^ < 50%); CI—Confidence Interval; NA—Not applicable. Abbreviations: AZT—Zidovudine; d4T—Stavudine; 3TC—Lamivudine; NVP—Nevirapine; EFV—Efavirenz.

**Table 5:** Relative mutation risk ratio (MRR) according to treatment regimen.

Treatment Regimen	MRR ^a^	95% CI ^b^	Std. Error	*p*-Value *
Drug-naive	1	-	-	-
AZT (or d4T) + 3TC + NVP	0.586	0.1670; 1.0058	0.2140	0.2301
AZT (or d4T) + 3TC + NVP (or EFV)	2.883	2.5829; 3.1839	0.1533	<0.00001
NVP (after birth) + AZT (first week of life)	3.754	3.3274; 4.1797	0.2174	<0.00001

^a^ MRR—Mutation Risk Ratio; ^b^ CI—Confidence Interval; * Z-test for means (H0: MRR = 1). Abbreviations: AZT—Zidovudine; d4T—Stavudine; 3TC—Lamivudine; NVP—Nevirapine; EFV—Efavirenz.

**Table 6:** Drug resistance mutation rate meta-regression adjusted for treatment, male ratio, subtype C ratio, origin of HIV sequences, region of study and participant group (I^2^ = 54.0% and R2 = 34.3%).

Index Specimen	Coefficient	Std. Error	*p*-Value *
**Overall**			
**Intercept**	16.28	5.17	0.0016
**Male (%)**	5.58	6.89	0.4183
**Subtype C**	−17.31	5.36	0.0012
**Treatment (AZT or d4T + 3TC + NVP as reference)**			
Drug-naïve	0.39	0.40	0.3229
AZT (or d4T) + 3TC+ NVP (or EFV)	1.95	0.60	0.0011
NVP (after birth) + AZT (first week of life)	0.86	0.54	0.1133
**Sequence origin (cells)**			
Plasma	−4.45	1.13	<0.0001
**Localization (Nampula/Pemba as reference)**			
Mozambique multicentric	1.21	0.47	0.0104
Maputo	2.23	0.71	0.0016
Maputo and Beira	−0.73	0.51	0.1505
**Participant group (Adult as reference)**			
Pregnant women	−0.14	0.38	0.7057
Neonates/Infants/Children	0.86	0.54	0.1133

* Z-test for means (H0: Coefficient = 0). AZT—Zidovudine; d4T—Stavudine; 3TC—Lamivudine; NVP—Nevirapine; Std. error—Standard error.

**Table 7:** Meta-analysis of viral load by study treatment.

Treatment Regimen	No. Studies	Viral Load (log_10_ HIV-1 RNA Copies)	95% CI ^a^	I^2^ (%)
Drug-naive	3	4.373	4.053; 4.694	86.5 *
AZT (or d4T) + 3TC + NVP	1	3.100	2.778; 3.422	NA

^a^ CI—Confidence Interval; * Chi-square *p*-value < 0.01 for heterogeneity (H0: I^2^ < 50%); NA—Not applicable; Abbreviations: AZT—Zidovudine; d4T—Stavudine; 3TC—Lamivudine; NVP—Nevirapine.

**Table 8:** Viral load meta-regression models adjusted for subtype C and treatment for model 1, and treatment and patient group for model 2.

Index Specimen	Studies	Coefficient	Std. Error	*p*-Value *
**Model 1**				
**Intercept**		21.13	4.31	<0.001
**Subtype C**		−17.03	4.36	<0.001
**Treatment (Drug-naïve as reference)**	3	-	-	-
AZT/d4T + 3TC + NVP	1	−2.24	0.32	<0.001
**Model 2**				
**Intercept**		4.60	0.18	<0.001
**Treatment (Drug-naïve as reference)**	3	-	-	-
AZT/d4T + 3TC + NVP	1	−1.50	0.27	<0.001
**Participant group (pregnant as reference)**	2	-	-	-
Adults	2	−0.35	0.21	0.098

* Z-test for means (H0: Coefficient = 0). AZT—Zidovudine; d4T—Stavudine; 3TC—Lamivudine; NVP—Nevirapine; Std. error—Standard error.

**Table 9:** CD4+ T-cell counts meta-regression adjusted for treatment and participant group.

Index Specimen	Studies	Coefficient	Std. Error	*p*-Value *
**Intercept**		374.00	35.24	<0.001
**Participant group (pregnant as reference)**	2	-	-	-
Adults	2	12.40	49.05	0.800
**Treatment (Drug-naive as reference)**	3	-	-	-
AZT/d4T + 3TC + NVP	1	244.00	64.06	<0.001

* Z-test for means (H0: Coefficient = 0). AZT—Zidovudine; d4T—Stavudine; 3TC—Lamivudine; NVP—Nevirapine; Std. error—Standard error.

**Table 10:** Prevalence of non-polymorphic and polymorphic HIV-1 reverse transcriptase and protease mutations in drug-naive and treated subjects.

Resistance Mutations	Naive, No (%) (n = 837)	Treated, No. (%) (n = 281)	*p*-Value *
**NRTI resistance mutations**			
K65R	0 (0)	3 (1.1)	0.002
M184V	9 (1.1)	17 (6.0)	<0.0001
**NNRTI resistance mutations**			
K103N	8 (0.96)	11 (3.9)	0.001
V106M	0 (0)	3 (1.1)	0.002
Y181C	2 (0.24)	21 (7.5)	<0.0001
G190A	5 (0.60)	8 (2.8)	0.002
H221Y	0 (0)	4 (1.4)	0.0005
**PI resistance mutations**			
Q58E	2 (0.24)	7 (2.49)	0.0003
G73S	0 (0)	3 (1.1)	0.002

* Z-test for pairwise comparison of proportions; No—Number.

## Data Availability

The data that support the findings of this study are available from the corresponding author, upon reasonable request.

## Supplementary Materials

The following supporting information can be downloaded at: https://www.mdpi.com/article/10.3390/v16121808/s1, Table S1: Details of the bibliographic search strategies used; Table S2: List of accession numbers of sequences retrieved from the GenBank (National Centre for Biotechnology Information) database based on the selected articles; Table S3: Mutations associated with resistance to protease inhibitors, reverse transcriptase inhibitors and integrase inhibitors in sequences included in this study; Figure S1: Drug resistance mutation rates (DRMR) in drug-naive individuals according to: (a) the region where the studies were conducted, (b) the types of samples collected for HIV sequencing, and (c) the participant group; Figure S2: Drug resistance mutation rates (DRMR) in patients on ART according to: (a) the region where the studies were conducted, (b) the types of samples collected for HIV sequencing, and (c) the group of participants; Figure S3: Drug resistance mutation rates (DRMR) in treated and untreated individuals; Figure S4: Logarithm of viral load in treated and untreated individuals.
