# Supplementary material for: HIV-1 Antiretroviral Drug Resistance in Mozambique: A Systematic Review and Meta-Analysis
Source: Viruses. 2024 Nov 21;16(12):1808. doi: 10.3390/v16121808 (PMC11680126; doi:10.3390/v16121808)
Supplement: Supplementary file 1 [file viruses-16-01808-s001.zip › viruses-3302445-supplementary.pdf]

Table S1- Details of the bibliographic search strategies used.

DATABASE	SEARCH STRATEGY
MEDLINE via PubMed	((HIV[tiab]) OR (AIDS[tiab]) OR (acquired immunodeficiency syndrome [tiab]) OR (human immunodeficiency virus[tiab])) AND (anti hiv agents[MeSH] OR Combination Antiretroviral Therapy[MeSH] OR Antiretroviral Therapy, Highly Active[MeSH] OR Anti-Retroviral drugs[MeSH] OR Antiviral drugs[MeSH:NoExp] OR ((anti) AND (hiv[tw])) OR antiretroviral*[tw] OR ((anti) AND (retroviral*[tw])) OR HAART[tw] OR ((anti) AND (acquired immunodeficiency[tw])) OR ((anti) AND (acquired immuno-deficiency[tw])) OR ((anti) AND (acquired immune-deficiency[tw])) OR ((anti) AND (acquired immun*) AND deficiency[tw])) AND Mozambique[tiab] AND (("drug resistance"[MeSH] OR resistance[Text Word] OR resistant[Text Word] "drug resistance"[Text Word] OR "drug resistant"[Text Word]) OR genotype)
B-On	AB (HIV OR AIDS) AND (therapy OR drug OR treatment OR antiretroviral) AND (resistant OR resistance OR HIVDR) AND (mutation OR genotype OR genetic OR SNP) AND AB Mozambique
SCOPUS	TITLE-ABS-KEY (hiv OR aids OR "human immunodeficiency virus" OR "acquired immunodeficiency syndrome") AND TITLE-ABS-KEY (mozambique) AND TITLE-ABS-KEY ("antiretroviral*" OR "antiretroviral drug" OR "antiretroviral therapy" OR "Highly Active Antiretroviral Therapy" OR "Anti-Retrovrial Agent" OR "Antiviral Agent" OR haart OR (anti AND hiv) OR (anti AND retroviral) OR (anti AND "acquired immunodeficiency") OR (anti AND "acquired immuno-deficiency") OR (anti AND "acquired immune-deficiency") OR (anti AND "acquired immun*" AND deficiency)) AND TITLE-ABS-KEY (resistan*)
COCHRANE	TOPICS: Infectious disease / HIV/AIDS

Table S2 - List of accession numbers of sequences retrieved from the GenBank (National Centre for Biotechnology Information) database based on the selected articles.

**Accession Numbers**			
	**Andreotti et al. [2009]**		
FJ750217 (plasma)	FJ750102 (plasma)	FJ749969 (breast milk)	FJ750085 (plasma)
FJ750216 (plasma)	FJ750101 (plasma)	FJ694840 (BMC)	FJ750084 (plasma)
FJ750215 (plasma)	FJ750100 (plasma)	FJ694839 (BMC)	FJ750083 (plasma)
FJ750214 (plasma)	FJ750099 (plasma)	FJ694838 (BMC)	FJ750082 (plasma)
FJ750213 (plasma)	FJ750098 (plasma)	FJ694837 (BMC)	FJ750081 (plasma)
FJ750212 (plasma)	FJ750097 (plasma)	FJ694836 (BMC)	FJ750080 (plasma)
FJ750211 (plasma)	FJ750096 (plasma)	FJ694835 (BMC)	FJ750079 (plasma)
FJ750210 (plasma)	FJ750095 (plasma)	FJ694834 (BMC)	FJ750078 (plasma)
FJ750209 (plasma)	FJ750094 (plasma)	FJ694833 (BMC)	FJ750077 (plasma)
FJ750208 (plasma)	FJ750093 (plasma)	FJ694832 (BMC)	FJ750076 (plasma)
FJ750207 (plasma)	FJ750092 (plasma)	FJ694831 (BMC)	FJ750075 (plasma)
FJ750206 (plasma)	FJ750091 (plasma)	FJ694830 (BMC)	FJ750074 (plasma)
FJ750205 (plasma)	FJ750090 (plasma)	FJ694829 (BMC)	FJ750073 (plasma)
FJ750204 (plasma)	FJ750089 (plasma)	FJ694828 (BMC)	FJ750072 (plasma)
FJ750203 (plasma)	FJ750088 (plasma)	FJ694827 (BMC)	FJ750071 (plasma)
FJ750202 (plasma)	FJ750067 (plasma)	FJ694826 (BMC)	FJ750070 (plasma)
FJ750201 (plasma)	FJ750066 (plasma)	FJ694825 (BMC)	FJ750069 (plasma)
FJ750200 (plasma)	FJ750064 (BMC[a])	FJ694824 (BMC)	FJ750068 (plasma)
FJ750199 (plasma)	FJ750063 (BMC)	FJ694823 (BMC)	FJ750036 (BMC)
FJ750198 (plasma)	FJ750062 (BMC)	FJ694822 (BMC)	FJ750035 (BMC)
FJ750197 (plasma)	FJ750061 (BMC)	FJ694821 (BMC)	FJ750034 (BMC)
FJ750196 (plasma)	FJ750060 (BMC)	FJ694820 (BMC)	FJ750033 (BMC)
FJ750195 (plasma)	FJ750059 (BMC)	FJ694819 (BMC)	FJ750032 (BMC)
FJ750194 (plasma)	FJ750058 (BMC)	FJ694818 (BMC)	FJ750031 (BMC)
FJ750193 (plasma)	FJ750057 (BMC)	FJ694817 (BMC)	FJ750030 (BMC)
FJ750192 (plasma)	FJ750056 (BMC)	FJ694816 (BMC)	FJ750029 (BMC)
FJ750191 (plasma)	FJ750055 (BMC)	FJ694815 (BMC)	FJ750028 (BMC)
FJ750190 (plasma)	FJ750054 (BMC)	FJ694814 (BMC)	FJ750027 (BMC)
FJ750168 (plasma)	FJ750053 (BMC)	FJ694813 (BMC)	FJ750026 (BMC)
FJ750167 (plasma)	FJ750052 (BMC)	FJ694794 (BMC)	FJ750025 (BMC)
FJ750166 (breast milk)	FJ750051 (BMC)	FJ750189 (plasma)	FJ750024 (BMC)
FJ750165 (breast milk)	FJ750050 (BMC)	FJ750188 (plasma)	FJ750023 (BMC)
FJ750164 (breast milk)	FJ750049 (BMC)	FJ750187 (plasma)	FJ750022 (BMC)
FJ750163 (breast milk)	FJ750048 (BMC)	FJ750186 (plasma)	FJ750021 (BMC)
FJ750162 (breast milk)	FJ750047 (BMC)	FJ750185 (plasma)	FJ750020 (BMC)
FJ750161 (breast milk)	FJ750046 (BMC)	FJ750184 (plasma)	FJ749991 (breast milk)
FJ750160 (breast milk)	FJ750045 (BMC)	FJ750183 (plasma)	FJ749990 (breast milk)
FJ750159 (breast milk)	FJ750044 (BMC)	FJ750182 (plasma)	FJ749989 (breast milk)
FJ750158 (breast milk)	FJ750043 (BMC)	FJ750181 (plasma)	FJ749988 (breast milk)
FJ750157 (breast milk)	FJ750042 (BMC)	FJ750180 (plasma)	FJ749987 (breast milk)
FJ750156 (breast milk)	FJ750041 (BMC)	FJ750179 (plasma)	FJ749986 (breast milk)
FJ750155 (breast milk)	FJ750040 (BMC)	FJ750178 (plasma)	FJ749985 (breast milk)
FJ750154 (breast milk)	FJ750039 (BMC)	FJ750177 (plasma)	FJ749984 (breast milk)
FJ750153 (breast milk)	FJ750038 (BMC)	FJ750176 (plasma)	FJ749983 (breast milk)
FJ750152 (breast milk)	FJ750037 (BMC)	FJ750175 (plasma)	FJ749982 (breast milk)
FJ750151 (breast milk)	FJ750019 (breast milk)	FJ750174 (plasma)	FJ749981 (breast milk)
FJ750150 (breast milk)	FJ750018 (breast milk)	FJ750173 (plasma)	FJ749980 (breast milk)
FJ750149 (breast milk)	FJ750017 (breast milk)	FJ750172 (plasma)	FJ749979 (breast milk)
FJ750148 (breast milk)	FJ750016 (breast milk)	FJ750171 (plasma)	FJ749978 (breast milk)
FJ750147 (breast milk)	FJ750015 (breast milk)	FJ750170 (plasma)	FJ749977 (breast milk)
FJ750146 (breast milk)	FJ750014 (breast milk)	FJ750169 (plasma)	FJ749976 (breast milk)
FJ750145 (breast milk)	FJ750013 (breast milk)	FJ750138 (breast milk)	FJ749975 (breast milk)
FJ750144 (breast milk)	FJ750012 (breast milk)	FJ750137 (breast milk)	FJ749974 (breast milk)
FJ750143 (breast milk)	FJ750011 (breast milk)	FJ750136 (breast milk)	FJ749973 (breast milk)
FJ750142 (breast milk)	FJ750010 (breast milk)	FJ750135 (breast milk)	FJ749972 (breast milk)
FJ750141 (breast milk)	FJ750009 (breast milk)	FJ750134 (breast milk)	FJ749971 (breast milk)
FJ750140 (breast milk)	FJ750008 (breast milk)	FJ750133 (breast milk)	FJ749970 (breast milk)
FJ750139 (breast milk)	FJ750007 (breast milk)	FJ750132 (breast milk)	FJ694812 (BMC)
FJ750117 (breast milk)	FJ750006 (breast milk)	FJ750131 (breast milk)	FJ694811 (BMC)
FJ750116 (breast milk)	FJ750005 (breast milk)	FJ750130 (breast milk)	FJ694810 (BMC)
FJ750115 (plasma)	FJ750004 (breast milk)	FJ750129 (breast milk)	FJ694809 (BMC)
FJ750114 (plasma)	FJ750003 (breast milk)	FJ750128 (breast milk)	FJ694808 (BMC)

FJ750113 (plasma)	FJ750002 (breast milk)	FJ750127 (breast milk)	FJ694807 (BMC)
FJ750112 (plasma)	FJ750001 (breast milk)	FJ750126 (breast milk)	FJ694806 (BMC)
FJ750111 (plasma)	FJ750000 (breast milk)	FJ750125 (breast milk)	FJ694805 (BMC)
FJ750110 (plasma)	FJ749999 (breast milk)	FJ750124 (breast milk)	FJ694804 (BMC)
FJ750109 (plasma)	FJ749998 (breast milk)	FJ750122 (breast milk)	FJ694803 (BMC)
FJ750108 (plasma)	FJ749997 (breast milk)	FJ750121 (breast milk)	FJ694802 (BMC)
FJ750107 (plasma)	FJ749996 (breast milk)	FJ750120 (breast milk)	FJ694801 (BMC)
FJ750106 (plasma)	FJ749995 (breast milk)	FJ750119 (breast milk)	FJ694800 (BMC)
FJ750105 (plasma)	FJ749994 (breast milk)	FJ750118 (breast milk)	FJ694799 (BMC)
FJ750104 (plasma)	FJ749993 (breast milk)	FJ750087 (plasma)	FJ694798 (BMC)
FJ750103 (plasma)	FJ749992 (breast milk)	FJ750086 (plasma)	FJ694797 (BMC)
FJ694796 (BMC)	FJ694795 (BMC)		
**Oliveira et al. [2012]**			
JQ670845 (GD)	JQ670831 (GB)	JQ670816 (GA)	JQ670802 (GA)
JQ670844 (GD)	JQ670830 (GB)	JQ670815 (GA)	JQ670801 (GA)
JQ670843 (GD)	JQ670829 (GB)	JQ670814 (GA)	JQ670800 (GA)
JQ670842 (GD)	JQ670828 (GB)	JQ670813 (GA)	JQ670799 (GA)
JQ670841 (GD)	JQ670827 (GA)	JQ670812 (GA)	JQ670798 (GA)
JQ670840 (GD)	JQ670826 (GA)	JQ670811 (GA)	JQ670797 (GA)
JQ670839 (GD)	JQ670825 (GA)	JQ670810 (GA)	JQ670796 (GA)
JQ670838 (GD)	JQ670824 (GA)	JQ670809 (GA)	JQ670795 (GA)
JQ670837 (GD)	JQ670823 (GA)	JQ670808 (GA)	JQ670794 (GA)
JQ670836 (GC)	JQ670822 (GA)	JQ670807 (GA)	JQ670793 (GA)
JQ670835 (GC)	JQ670821 (GA)	JQ670806 (GA)	JQ670792 (GA)
JQ670834(GC)	JQ670820 (GA)	JQ670805 (GA)	JQ670791 (GA)
JQ670833 (GB)	JQ670819 (GA)	JQ670804 (GA)	
JQ670832 (GB)	JQ670817 (GA)	JQ670803 (GA)	
JQ670788 (GA)	JQ670789 (GA)	JQ670790 (GA)	
**Lahuerta et al. [2008]**			
EF407751	EF407739	EF407727	EF407715
EF407750	EF407738	EF407726	EF407714
EF407749	EF407737	EF407725	EF407713
EF407748	EF407736	EF407724	EF407712
EF407747	EF407735	EF407723	EF407711
EF407746	EF407734	EF407722	EF407710
EF407745	EF407733	EF407721	EF407709
EF407744	EF407732	EF407720	EF407708
EF407743	EF407731	EF407719	EF407707
EF407742	EF407730	EF407718	EF407706
EF407741	EF407729	EF407717	EF407705
EF407740	EF407728	EF407716	EF407704
EF407703	EF407702	EF407701	
**Micek et al. [2014]**			
KJ395315	KJ395324	KJ395333	KJ395342
KJ395316	KJ395325	KJ395334	KJ395343
KJ395317	KJ395326	KJ395335	KJ395344
KJ395318	KJ395327	KJ395336	KJ395345
KJ395319	KJ395328	KJ395337	KJ395346
KJ395320	KJ395329	KJ395338	KJ395347
KJ395321	KJ395330	KJ395339	
KJ395322	KJ395331	KJ395340	
KJ395323	KJ395332	KJ395341	
**Bila et al. [2013]**			
JX507920	JX507892	JX507864	JX507836
JX507919	JX507891	JX507863	JX507835
JX507918	JX507890	JX507862	JX507834
JX507917	JX507889	JX507861	JX507833
JX507916	JX507888	JX507860	JX507832
JX507915	JX507887	JX507859	JX507831
JX507914	JX507886	JX507858	JX507830
JX507913	JX507885	JX507857	JX507829
JX507912	JX507884	JX507856	JX507828
JX507911	JX507883	JX507855	JX507827
JX507910	JX507882	JX507854	JX507826
JX507909	JX507881	JX507853	JX507825
JX507908	JX507880	JX507852	JX507824
JX507907	JX507879	JX507851	JX507823
JX507906	JX507878	JX507850	JX507822

JX507905	JX507877	JX507849	JX507821
JX507904	JX507876	JX507848	JX507820
JX507903	JX507875	JX507847	JX507819
JX507902	JX507874	JX507846	JX507818
JX507901	JX507873	JX507845	JX507817
JX507900	JX507872	JX507844	JX507816
JX507899	JX507871	JX507843	JX507815
JX507898	JX507870	JX507842	JX507814
JX507897	JX507869	JX507841	JX507813
JX507896	JX507868	JX507840	JX507812
JX507895	JX507867	JX507839	JX507811
JX507894	JX507866	JX507838	JX507810
JX507893	JX507865	JX507837	JX507809
**Vubil et al. [2016]**			
KT729409	KT729433	KT729457	KT729481
KT729410	KT729434	KT729458	KT729482
KT729411	KT729435	KT729459	KT729483
KT729412	KT729436	KT729460	KT729484
KT729413	KT729437	KT729461	KT729485
KT729414	KT729438	KT729462	KT729486
KT729415	KT729439	KT729463	KT729487
KT729416	KT729440	KT729464	KT729488
KT729417	KT729441	KT729465	KT729489
KT729418	KT729442	KT729466	KT729490
KT729419	KT729443	KT729467	KT729491
KT729420	KT729444	KT729468	KT729492
KT729421	KT729445	KT729469	KT729493
KT729422	KT729446	KT729470	KT729494
KT729423	KT729447	KT729471	KT729495
KT729424	KT729448	KT729472	KT729496
KT729425	KT729449	KT729473	KT729497
KT729426	KT729450	KT729474	KT729498
KT729427	KT729451	KT729475	KT729499
KT729428	KT729452	KT729476	KT729500
KT729429	KT729453	KT729477	KT729501
KT729430	KT729454	KT729478	KT729502
KT729431	KT729455	KT729479	KT729503
KT729432	KT729456	KT729480	
**Bellocchi et al. [2005]**			
GU199582	GU199567	GU199552	GU199537
GU199581	GU199566	GU199551	GU199536
GU199580	GU199565	GU199550	GU199535
GU199579	GU199564	GU199549	GU199534
GU199578	GU199563	GU199548	GU199533
GU199577	GU199562	GU199547	GU199532
GU199576	GU199561	GU199546	GU199531
GU199575	GU199560	GU199545	GU199530
GU199574	GU199559	GU199544	GU199529
GU199573	GU199558	GU199543	GU199528
GU199572	GU199557	GU199542	GU199527
GU199571	GU199556	GU199541	GU199526
GU199570	GU199555	GU199540	GU199525
GU199569	GU199554	GU199539	GU199524
GU199568	GU199553	GU199538	
**Parreira et al. [2006]**			
AM071454	AM071443	AM071432	AM071421
AM071453	AM071442	AM071431	AM071420
AM071452	AM071441	AM071430	AM071419
AM071451	AM071440	AM071429	AM071418
AM071450	AM071439	AM071428	AM071417
AM071449	AM071438	AM071427	AM071416
AM071448	AM071437	AM071426	AM071415
AM071447	AM071436	AM071425	AM071414
AM071446	AM071435	AM071424	AM071413
AM071445	AM071434	AM071423	AM071412
AM071444	AM071433	AM071422	
**Bártolo et al. [2009]**			
EF071942	DQ660104	DQ660057	DQ660010

EF071941	DQ660103	DQ660056	DQ660009
EF071940	DQ660102	DQ660055	DQ660008
EF071938	DQ660100	DQ660054	DQ660007
EF071937	DQ660099	DQ660053	DQ660006
EF071936	DQ660098	DQ660052	DQ660005
EF071935	DQ660097	DQ660051	DQ660004
EF071934	DQ660096	DQ660050	DQ660003
EF071933	DQ660095	DQ660049	DQ660002
EF071932	DQ660094	DQ660048	DQ660001
EF071931	DQ660093	DQ660047	DQ660000
EF071930	DQ660092	DQ660046	DQ659999
DQ660139	DQ660091	DQ660045	DQ659998
DQ660138	DQ660090	DQ660044	DQ659997
DQ660137	DQ660089	DQ660043	DQ659996
DQ660136	DQ660088	DQ660042	DQ659995
DQ660135	DQ660087	DQ660041	DQ659994
DQ660134	DQ660086	DQ660040	DQ659993
DQ660133	DQ660085	DQ660039	DQ659992
DQ660132	DQ660084	DQ660038	DQ659991
DQ660131	DQ660083	DQ660037	DQ659990
DQ660130	DQ660082	DQ660036	DQ659989
DQ660129	DQ660081	DQ660035	DQ659988
DQ660128	DQ660080	DQ660034	DQ659987
DQ660127	DQ660079	DQ660033	DQ659986
DQ660126	DQ660078	DQ660032	DQ659985
DQ660125	DQ660077	DQ660031	DQ659984
DQ660124	DQ660076	DQ660030	DQ659983
DQ660123	DQ659981	DQ660029	DQ659982
DQ660122	DQ660075	DQ660028	DQ659980
DQ660121	DQ660074	DQ660027	DQ659979
DQ660120	DQ660073	DQ660026	DQ659978
DQ660119	DQ660072	DQ660025	DQ659977
DQ660118	DQ660071	DQ660024	DQ659976
DQ660117	DQ660070	DQ660023	DQ659975
DQ660116	DQ660069	DQ660022	DQ659974
DQ660115	DQ660068	DQ660021	DQ659973
DQ660114	DQ660067	DQ660020	DQ659972
DQ660113	DQ660066	DQ660019	DQ659971
DQ660112	DQ660065	DQ660018	DQ659970
DQ660111	DQ660064	DQ660017	DQ659969
DQ660110	DQ660063	DQ660016	DQ659968
DQ660109	DQ660062	DQ660015	DQ659967
DQ660108	DQ660061	DQ660014	DQ659966
DQ660107	DQ660060	DQ660013	DQ659965
DQ660106	DQ660059	DQ660012	
DQ660105	DQ660058	DQ660011	
**Vaz et al. [2012]**			
KT365514	KT365486	KT365458	KT365430
KT365513	KT365485	KT365457	KT365429
KT365512	KT365484	KT365456	KT365428
KT365511	KT365483	KT365455	KT365427
KT365510	KT365482	KT365454	KT365426
KT365509	KT365481	KT365453	KT365425
KT365508	KT365480	KT365452	KT365424
KT365507	KT365479	KT365451	KT365423
KT365506	KT365478	KT365450	KT365422
KT365505	KT365477	KT365449	KT365421
KT365504	KT365476	KT365448	KT365420
KT365503	KT365475	KT365447	KT365419
KT365502	KT365474	KT365446	KT365418
KT365501	KT365473	KT365445	KT365417
KT365500	KT365472	KT365444	KT365416
KT365499	KT365471	KT365443	KT365415
KT365498	KT365470	KT365442	KT365414
KT365497	KT365469	KT365441	KT365413
KT365496	KT365468	KT365440	KT365412
KT365495	KT365467	KT365439	KT365411
KT365494	KT365466	KT365438	KT365410

KT365493	KT365465	KT365437	KT365409
KT365492	KT365464	KT365436	KT365408
KT365491	KT365463	KT365435	KT365407
KT365490	KT365462	KT365434	KT365406
KT365489	KT365461	KT365433	KT365405
KT365488	KT365560	KT365432	KT365404
KT365487	KT365459	KT365431	KT365403
**Abreu et al. [2008]**			
KF927434	KF927414	KF927395	KF927376
KF927433	KF927413	KF927394	KF927375
KF927432	KF927412	KF927393	KF927374
KF927431	KF927411	KF927392	KF927373
KF927430	KF927410	KF927391	KF927372
KF927429	KF927409	KF927390	KF927371
KF927428	KF927408	KF927389	KF927370
KF927427	KF927407	KF927388	KF927369
KF927426	KF927406	KF927387	KF927368
KF927425	KF927405	KF927386	KF927367
KF927424	KF927404	KF927385	KF927366
KF927422	KF927403	KF927384	KF927365
KF927421	KF927402	KF927383	KF927364
KF927420	KF927401	KF927382	KF927363
KF927419	KF927400	KF927381	KF927362
KF927418	KF927399	KF927380	KF927361
KF927417	KF927398	KF927379	KF927360
KF927416	KF927397	KF927378	KF927359
KF927415	KF927396	KF927377	KF927358

[a] BMC – Breast Milk Cells

Table S3 - Mutations associated with resistance to protease inhibitors, reverse transcriptase inhibitors and integrase inhibitors in sequences included in this study[a].

Reverse transcriptase (RT)			Protease (PR)			Integrase (IN)		
**Mutation**	**Naïve, No (%) (n=837)**	**Treated, No. (%) (n=281)**	**Mutation**	**Naïve, No (%) (n=837)**	**Treated, No. (%) (n=281)**	**Mutation**	**Naïve, No (%) (n=837)**	**Treated, No. (%) (n=281)**
**NRTI**								
F116Y	1 (0.1)	0 (0)	M46I/V	2 (0.2)	1 (0.4)	G163R	0 (0)	1 (0.4)
K70Q/R	5 (0.6)	1 (0.4)	I84V	1 (0.1)	0 (0)	E157Q	0 (0)	1 (0.4)
V75A	1 (0.1)	0 (0)	N83D	1 (0.1)	0 (0)	T97A	1 (0.1)	0 (0)
M184V	9 (1.1)	17 (6.0)	L10F	3 (0.4)	0 (0)	G140R	1 (0.1)	0 (0)
L210W	1 (0.1)	0 (0)	K43T	0 (0)	1 (0.4)			
T215A/F/I/Y	10 (1.2)	0 (0)	Q58E	2 (0.2)	7 (2.5)			
K219E/Q	3 (0.4)	1 (0.4)	K20T	3 (0.4)	0 (0)			
M41L	4 (0.5)	1 (0.4)	G73S	0 (0)	3 (1.1)			
D67N	3 (0.4)	0 (0)	G48R	0 (0)	1 (0.4)			
A62V	0 (0)	2 (0.7)	I50L	1 (0.1)	0 (0)			
K65R/N	0 (0)	4 (1.4)	V32E/G	2 (0.2)	0 (0)			
L74V/I	0 (0)	3 (1.1)	L89V	1 (0.1)	0 (0)			
			T74P	1 (0.1)	0 (0)			
**NNRTI**			L90M	1 (0.1)	0 (0)			
A98G	3 (0.4)	3 (1.1)	I50F	1 (0.1)	0 (0)			
P236L	1 (0.1)	0 (0)	I84M	1 (0.1)	0 (0)			
E138A	67 (8.0)	20 (7.1)						
Y181C	4 (0.5)	21 (7.5)						
K103N	8 (1.0)	11 (3.9)						
V106I	2 (0.2)	0 (0)						
V108I	1 (0.1)	2 (0.7)						
K101E/H	4 (0.5)	2 (0.7)						
V179D/E/T	8 (1.0)	4 (1.4)						
G190A	5 (0.6)	8 (2.8)						
Y188C/F	0 (0)	2 (0.7)						
M230I	1 (0.1)	2 (0.7)						
L100F	0 (0)	1 (0.4)						
K238N	0 (0)	1 (0.4)						
L234I	0 (0)	1 (0.4)						
P225H	2 (0.2)	0 (0)						
V106A/M	0 (0)	5 (1.8)						
H221Y	0 (0)	4 (1.4)						

[a]According to the Stanford HIV Drug Resistance Database (https://hivdb.stanford.edu/hivdb/by-sequences/)

a)

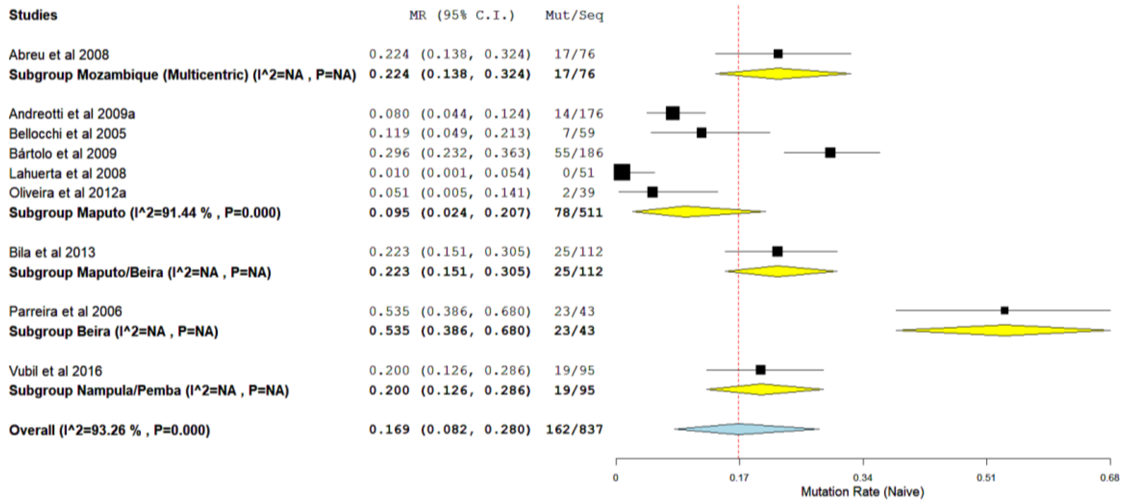

b)

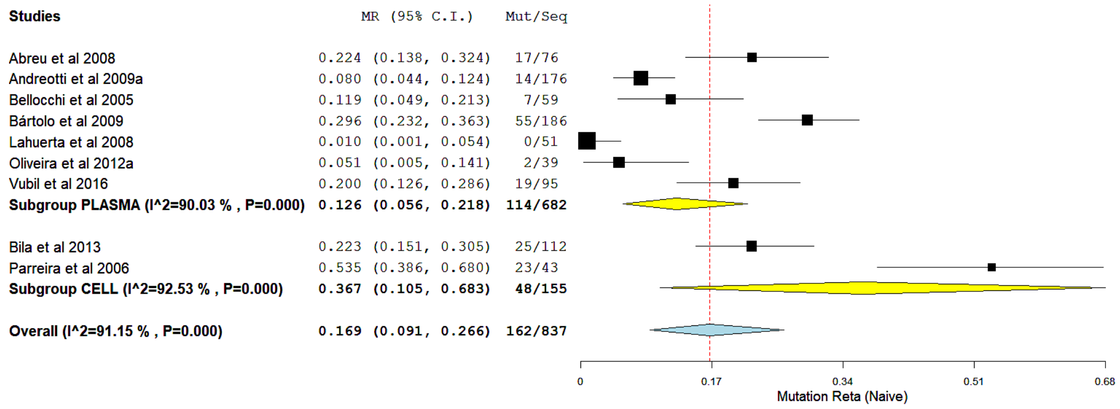

c)

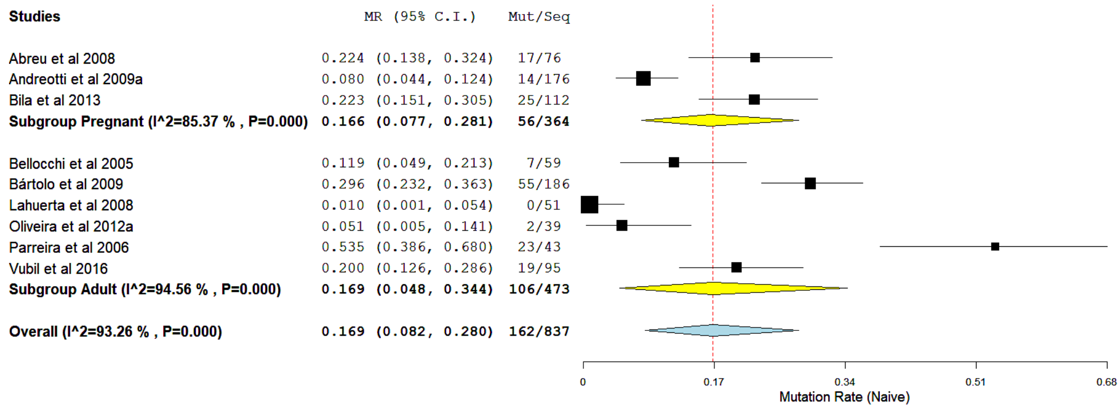

Figure S1- Drug resistance mutation rates (DRMR) in drug-naive individuals according to: a) the region where the studies were conducted, b) the types of samples collected for HIV sequencing, and c) the participant group. I2 represents the heterogeneity index, and the overall statistical significance of homogeneity was calculated using the χ2 test. The area of the squares represent the sample size, the continuous horizontal lines and the

width of the diamonds represent the 95% confidence interval. The center of the yellow diamond indicates the pooled subgroup estimates, while the center of the blue diamond and the vertical red dotted line indicate the overall pooled estimate. The studies included in the forest plot contributed a total of 837 pol gene sequences.

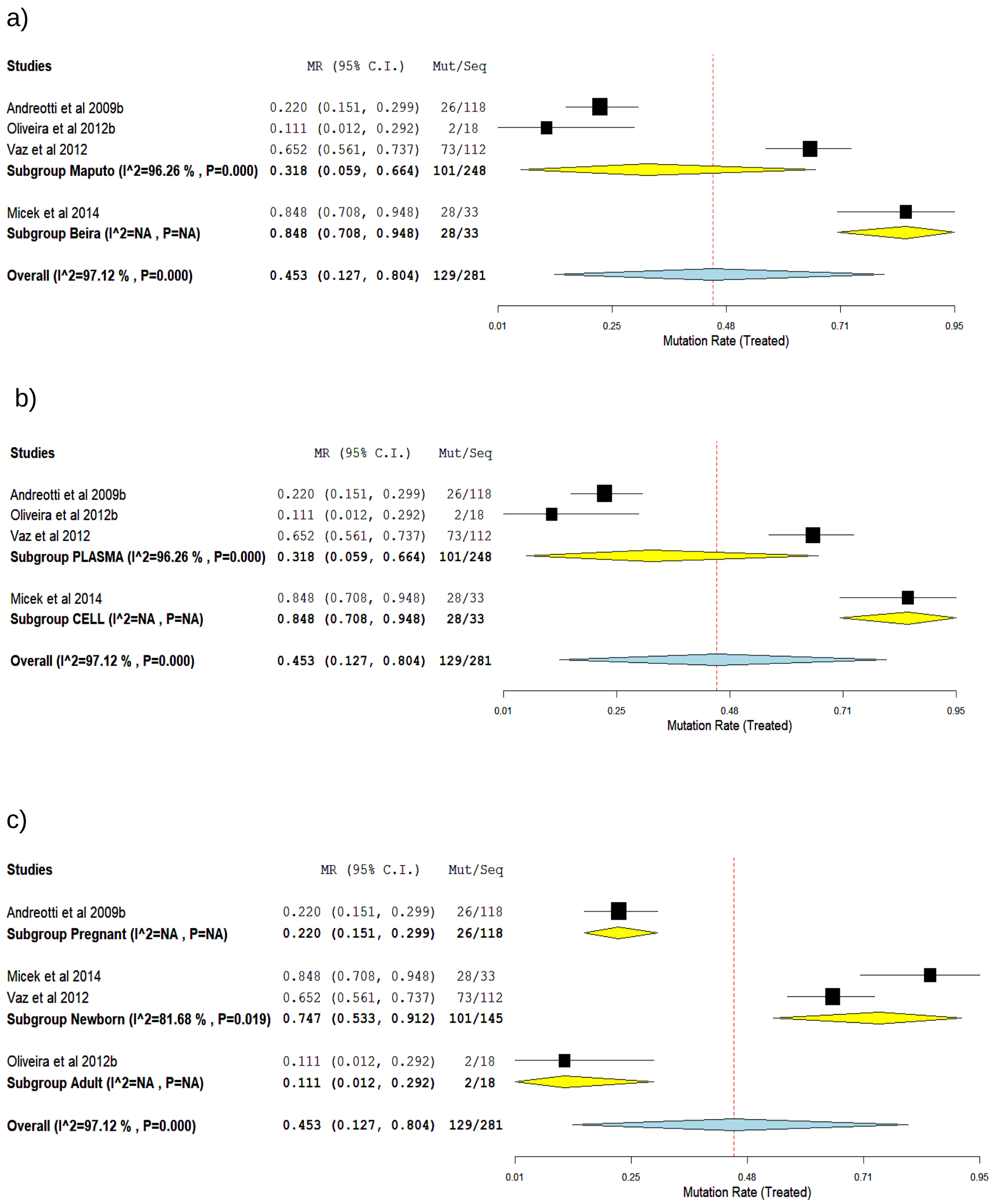

Figure S2 - Drug resistance mutation rates (DRMR) in patients on ART according to: a) the region where the studies were conducted, b) the types of samples collected for HIV sequencing, and c) the group of participants. I2 represents the heterogeneity index, and

the overall statistical significance of homogeneity was calculated using the χ2 test. The area of the squares represent the sample size, the continuous horizontal lines and the width of the diamonds represent the 95% confidence interval. The center of the yellow diamond indicates the pooled subgroup estimates, while the center of the blue diamond and the vertical red dotted line indicate the overall pooled estimate. The studies included in the forest plot contributed a total of 281 pol gene sequences.

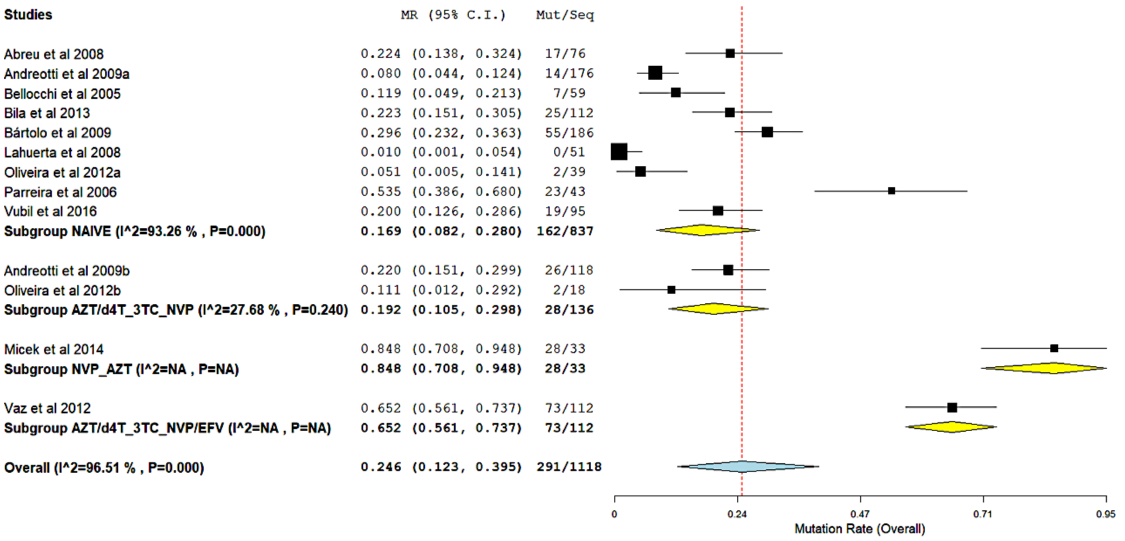

Figure S3 - Drug resistance mutation rates (DRMR) in treated and untreated individuals. The studies included in the forest plot contributed a total of 1118 pol gene sequences.

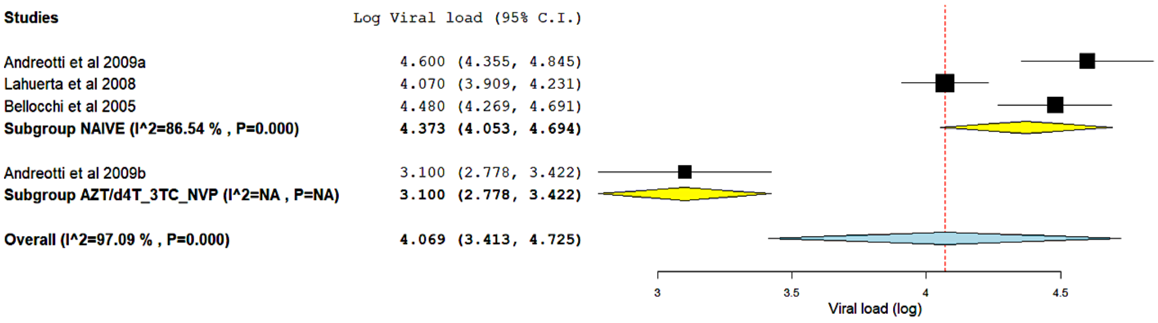

Figure S4 - Logarithm of viral load in treated and untreated individuals. I2 represents the heterogeneity index and the overall statistical significance of homogeneity was calculated using the χ2 test. The area of the squares represents the sample size, the continuous horizontal lines and the width of the diamonds represent the 95% confidence interval. The center of the yellow diamond indicates the pooled subgroup estimates,

while the center of the blue diamond and the vertical red dotted line indicate the overall pooled estimate.
